# Mitochondrial mass governs the extent of human T cell senescence

**DOI:** 10.1111/acel.13067

**Published:** 2019-12-02

**Authors:** Lauren A. Callender, Elizabeth C. Carroll, Emilia A. Bober, Arne N. Akbar, Egle Solito, Sian M. Henson

**Affiliations:** ^1^ Translational Medicine and Therapeutics William Harvey Research Institute Barts and The London School of Medicine and Dentistry Queen Mary University of London London UK; ^2^ Department of Life Sciences IT Sligo Sligo Ireland; ^3^ Division of Infection and Immunity Department of Immunology University College London London UK

**Keywords:** aging, metabolism, mitochondria, senescence, T cell

## Abstract

The susceptibility of human CD4^+^ and CD8^+^ T cells to senesce differs, with CD8^+^ T cells acquiring an immunosenescent phenotype faster than the CD4^+^ T cell compartment. We show here that it is the inherent difference in mitochondrial content that drives this phenotype, with senescent human CD4^+^ T cells displaying a higher mitochondrial mass. The loss of mitochondria in the senescent human CD8^+^ T cells has knock‐on consequences for nutrient usage, metabolism and function. Senescent CD4^+^ T cells uptake more lipid and glucose than their CD8^+^ counterparts, leading to a greater metabolic versatility engaging either an oxidative or a glycolytic metabolism. The enhanced metabolic advantage of senescent CD4^+^ T cells allows for more proliferation and migration than observed in the senescent CD8^+^ subset. Mitochondrial dysfunction has been linked to both cellular senescence and aging; however, it is still unclear whether mitochondria play a causal role in senescence. Our data show that reducing mitochondrial function in human CD4^+^ T cells, through the addition of low‐dose rotenone, causes the generation of a CD4^+^ T cell with a CD8^+^‐like phenotype. Therefore, we wish to propose that it is the inherent metabolic stability that governs the susceptibility to an immunosenescent phenotype.

## Graphical abstract

Human CD8^+^ EMRA T cells are acquired at a faster rate owing to their lower mitochondrial content. This leads to impaired nutrient uptake by CD8^+^ EMRA T cell, which impacts their function.

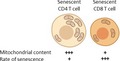

## 1 INTRODUCTION

The human immune system functionality declines with age in a process referred to as immunosenescence. The functional outcomes of this process include the compromised ability of older individuals to mount protective immune responses against both previously encountered and new pathogens (Akbar, Henson, & Lanna, 2016). Additionally, there is a marked decrease in vaccine efficacy in these populations. While these age‐associated alterations arise from defects in different leucocyte populations, the dysfunction is most profound in T cell subsets (Akbar et al., 2016). Furthermore, aging is associated with a chronic low‐grade inflammatory state, termed inflammaging (Franceschi et al., 2000), and mediates an important role in a range of age‐related degenerative pathologies (Baker et al., 2011). The source of this inflammation has yet to be defined. Senescent T cells are found to accumulate with age and represent a likely contributor to this inflammatory state that is observed during aging (Akbar et al., 2016).

Primary human senescent T cells are a highly differentiated subset of cells found within the CD27^−^CD28^−^ population (Parish, Wu, & Effros, 2010). This subset can be further characterized on the basis of CD45RA expression, with highly differentiated T cells that re‐express CD45RA identified as the senescent T cell population (EMRA; effector memory CD45RA re‐expressing T cells). They display multiple characteristics of senescence including a low proliferative activity, high levels of DNA damage and loss of telomerase activity (Henson et al., 2014). However, the response patterns of CD4^+^ and CD8^+^ T cells to aging differ, with CD8^+^ T cells being more susceptible to both phenotypic and functional changes during aging (Czesnikiewicz‐Guzik et al., 2008). The CD8^+^ EMRA T cell subset accumulates in higher proportions with age and is more prevalent following in vitro culture than the CD4^+^ EMRAs (Czesnikiewicz‐Guzik et al., 2008). The cause of this difference has been suggested to be due to the differing homeostatic mechanisms and an increased gene expression instability of regulatory cell surface molecules in the CD8^+^ EMRA subset (Czesnikiewicz‐Guzik et al., 2008). We would like to postulate an alternate view that the metabolic versatility seen in CD4^+^ T cells confers a metabolic advantage to the CD4 EMRA subset allowing these T cells to better withstand the intrinsic or extrinsic effects governing differentiation.

Metabolic examination of CD4^+^ and CD8^+^ T cells suggests that their metabolic programming allows differential immunological functions to be performed. We demonstrate here that CD4^+^ T cells have a greater mitochondrial mass and are consistently more oxidative than CD8^+^ T cells, allowing them to sustain effector function. Whereas the metabolic programs that prioritize rapid biosynthesis such as glycolysis are favoured by CD8^+^ T cells, allowing for faster growth and proliferative rates (Cao, Rathmell, & Macintyre, 2014). We have previously shown that CD8^+^ EMRA T cells display impaired mitochondrial function (Henson et al., 2014) but are still unclear as to whether CD4^+^ EMRA T cells also exhibit mitochondrial dysfunction. We provide evidence that this is not the case, and CD4^+^ EMRA T cells have fitter, healthier mitochondria that are better able to meet the energy requirements of the CD4^+^ EMRA subset. Therefore, we propose that it is the inherent metabolic stability that governs the susceptibility to an immunosenescent phenotype.

## 2 RESULTS

### 2.1 Human CD4^+^ EMRA T cell development at a slower rate due to their higher mitochondrial content

**Figure 1:**
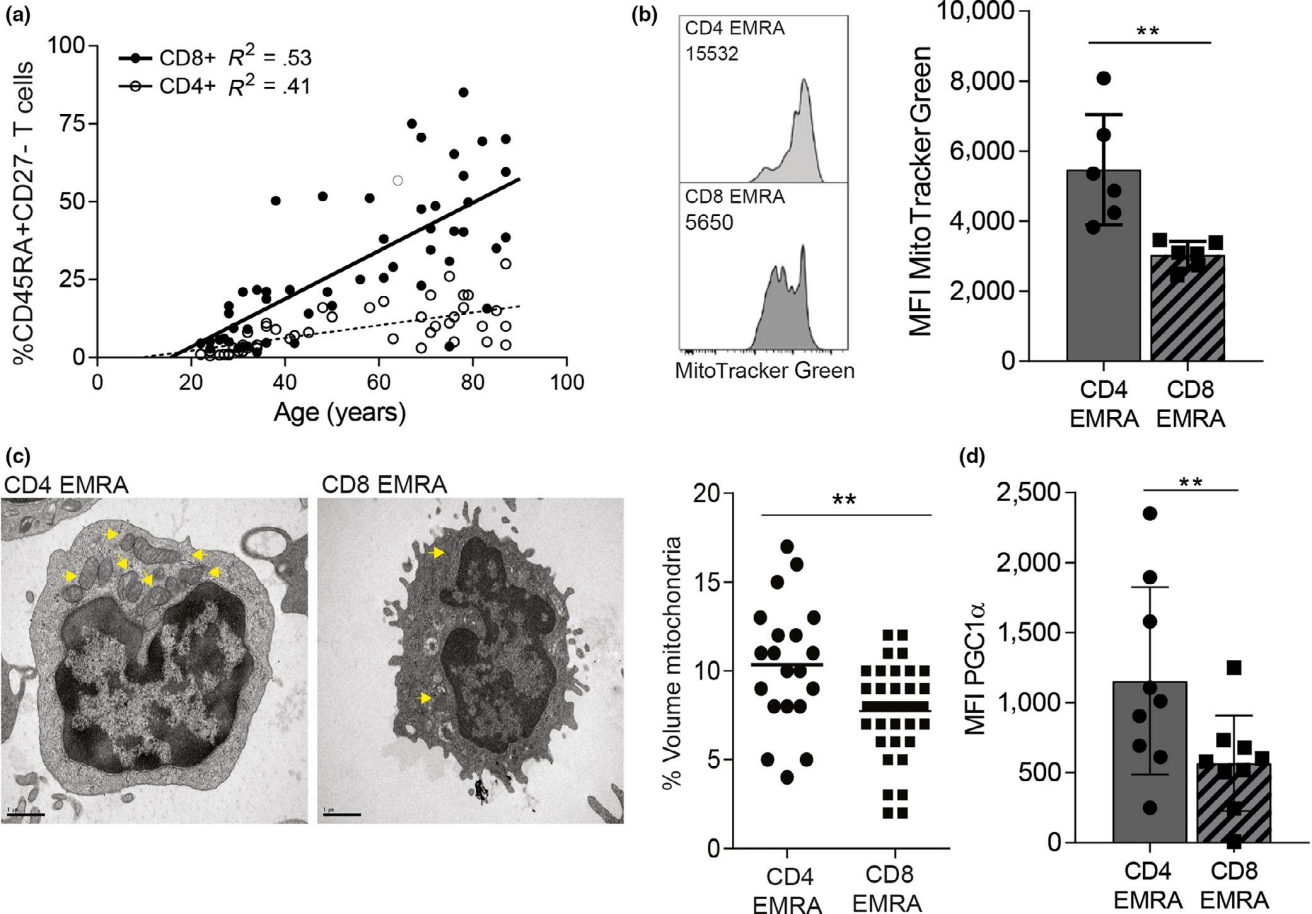
Human CD4^+^ EMRA T cells are acquired at a slower rate owing to a higher degree of mitochondrial content. (a) The accumulation of senescent CD4^+^ and CD8^+^ T cells with age defined by the markers CD45RA and CD27. (b) Representative flow cytometry plots from middle‐aged donors and cumulative graphs of MitoTracker Green staining in CD4^+^ and CD8^+^ EMRA T cells analysed directly ex vivo. Data expressed as mean ± *SEM* of six donors. (c) Electron microscope images of CD4^+^ and CD8^+^ EMRA T cells imaged directly ex vivo from middle‐aged donors*.* Yellow arrows mark mitochondria. Graph shows the percentage by cell volume of mitochondria in senescent T cell subsets determined by a point‐counting grid method from 20 different electron microscope images. (d) PGC1α expression in CD45RA/CD27‐defined EMRA T cell subsets from middle‐aged donors. Data expressed as mean ± *SEM* of nine donors. *p*‐values were calculated using a *t* test. ** *p* < .01

Human T cells can be subdivided into four populations on the basis of their relative surface expression of CD45RA and CD27 molecules (Figure S1a). The four subsets are defined as naïve (N; CD45RA^+^CD27^+^), central memory (CM; CD45RA^−^CD27^+^), effector memory (EM; CD45RA^−^CD27^−^) and effector memory T cells that re‐express CD45RA (EMRA; CD45RA^+^CD27^−^). We and others have demonstrated that the EMRA population exhibit numerous characteristics of senescence (Appay, Lier, Sallusto, & Roederer, 2008; Di Mitri et al., 2011; Henson et al., 2014); indeed, it has also been known for some time that CD4^+^ EMRA T cells senesce at a slow rate than their CD8^+^ counterparts (Figure 1a). It was thought that the difference in EMRA accumulation was due to their differing cytokine stabilities (Czesnikiewicz‐Guzik et al., 2008); however, we now demonstrate that it is a difference in mitochondrial mass between CD4^+^ and CD8^+^ EMRAs that governs the rate at which they develop.

Using MitoTracker Green, a mitochondrial‐specific dye that binds the mitochondrial membranes independently of mitochondrial membrane potential (MMP), we found the CD4^+^ EMRA subset isolated from middle‐aged donors (av. age 41 years ± 5) to have a significantly higher mitochondrial mass than CD8^+^ EMRAs, nearly double the amount of mitochondrial content (Figure 1b). The CD4^+^ EMRA subset retains their mitochondrial content compared to earlier less differentiated subsets (Figure S2a), whereas the CD8^+^ EMRAs do not (Henson et al., 2014). This was also borne out when the EMRA subsets were examined ex vivo by electron microscopy. We observed significantly fewer mitochondrial in the CD8^+^ EMRA compartment when compared to the CD4^+^ EMRA fraction using a point‐counting method (Figure 1c). Furthermore, when we investigated the expression of PGC1α (peroxisome proliferator‐activated receptor gamma coactivator 1‐alpha), the key regulator of mitochondrial biogenesis, the CD4^+^ EMRA subset showed significantly higher ex vivo levels of this marker than the CD8^+^ EMRAs (Figure 1d). This phenomenon was found to be independent of chronological age, as the mitochondrial content of CD4^+^ and CD8^+^ EMRA T cells isolated from older individuals (av. age 71 ± 3) was the same as that of younger individuals (Figure S2b,c). Collectively, our results demonstrate that senescent CD4^+^ T cells have increased mitochondrial mass in comparison with their CD8^+^ counterparts.

### 2.2 Distinct mitochondrial functions in CD4^+^ and CD8^+^ EMRA subsets

**Figure 2:**
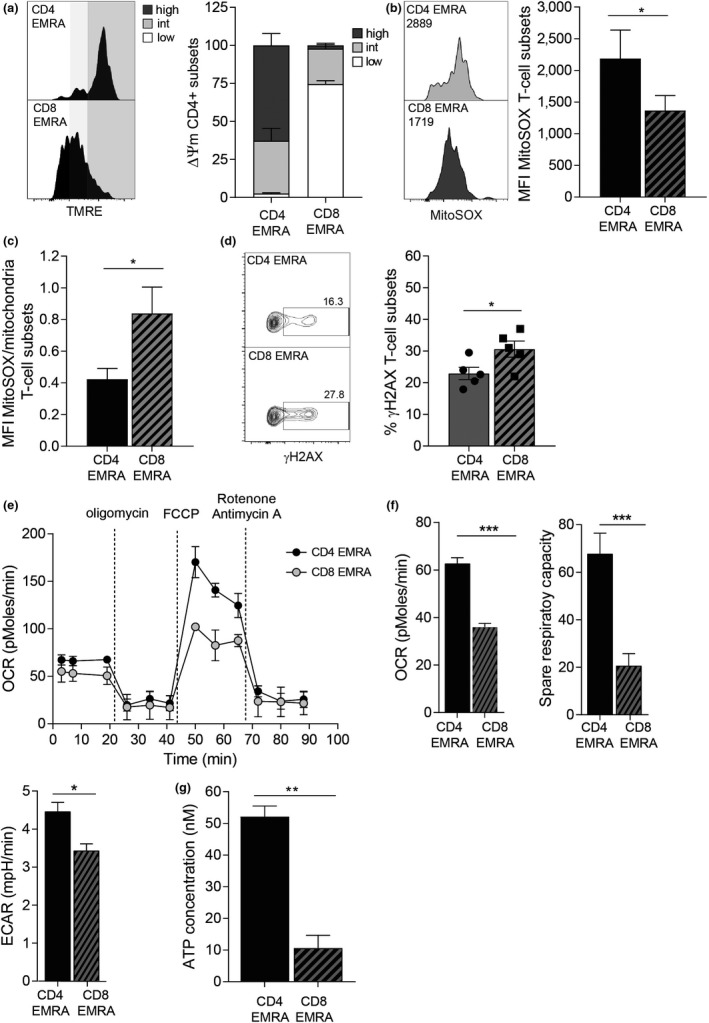
Mitochondrial dysfunction is observed in CD8^+^ but not CD4^+^ EMRA T cell subsets. (a) Representative flow cytometry plots and cumulative graphs of TMRE staining from middle‐aged donors showing membrane potential in CD45RA/CD27 T cell subsets directly ex vivo defined showing the percentage of cortactin‐positive (a) CD4^+^ and (b) CD8^+^ T cells analysed directly ex vivo. Data expressed as mean ± *SEM* of six donors. (b) Mitochondrial ROS measured using MitoSOX by flow cytometry in CD4^+^ and CD8^+^ EMRA T cells from middle‐aged donors. Data expressed as mean ± *SEM* of six donors. (c) Mitochondrial ROS production expressed as a ratio of mitochondrial mass. Calculated from data shown in Figures 1b and 2. (d) γH2AX expression as determined by flow cytometry in CD45RA/CD27‐defined T cell subsets directly ex vivo from middle‐aged donors; the graph shows the mean ± *SEM* for five donors. (e) Oxygen consumption rates (OCR) of the EMRA CD4^+^ and CD8^+^ T cell subsets from middle‐aged donors were measured following a 15‐min stimulation with 0.5 µg/ml anti‐CD3 and 5 ng/ml IL‐2; the cells were then subjected to a metabolic stress test using the indicated mitochondrial inhibitors. Data are representative of four independent experiments. (f) The basal OCR, extracellular acidification rate (ECAR) and spare respiratory capacity were measured following a 15‐min stimulation with 0.5 µg/ml anti‐CD3 and 5 ng/ml IL‐2. Graphs show the mean ± *SEM* for four donors. (g) ATP concentration in EMRA T cell subsets from middle‐aged donors, graphs show the mean ± *SEM* for five donors. *p*‐values were calculated using a *t* test. *p < .05, **p < .01, and ***p < .005

The increased mitochondrial mass seen in the CD4^+^ EMRA subsets suggests they may exhibit distinct mitochondrial functions compared to the CD8^+^ EMRAs. Indeed, using TMRE, which measures mitochondrial transmembrane potential, we found the CD4^+^ EMRAs had a higher proportion of hyperpolarized mitochondria than the CD8^+^ EMRA subset, which displayed a hypopolarized phenotype (Figures 2a and S3a). The mitochondrial membrane potential provides the charge gradient required for Ca^2+^ sequestration and the regulation of reactive oxygen species (ROS) production. Cell stress causes a dysregulation in the mitochondrial membrane potential, with hyperpolarization resulting in the production of excess ROS leading to oxidative stress. While a state of hypopolarization is also harmful, as low amounts of ROS cause reductive stress, which is as detrimental to homeostasis as oxidative stress (Zorova et al., 2018).

As hyperpolarized mitochondria can be a source of ROS that can potentiate senescence (Cui, Kong, & Zhang, 2012), we next measured mitochondrial ROS production using MitoSOX. We found the amount of ROS to be significantly higher in CD4^+^ EMRAs (Figures 2b and S3b); again, the observed differences were found to be independent of donor age (Figure S3c,d). However, both EMRA subsets produced significantly more ROS than their less differentiated counterparts (data not shown). However, the increased ROS production seen in CD4^+^ EMRAs was neutralized owing to the higher mitochondrial mass, meaning that CD8^+^ EMRA T cells produced more ROS per mitochondria that can potentially enhance their senescent phenotype (Figure 2c). Furthermore, increased ROS can also cause DNA damage and the activation of the DNA damage response, elevated during senescence. The examination of phosphorylated H2AX (γH2AX), a member of the histone H2A family that is part of the DNA damage response, in EMRAs revealed that the CD8^+^ EMRA subset displayed a higher level of this marker compared to CD4^+^ EMRAs (Figure 2d). However, both EMRA subsets express the highest amount of DNA damage compared to their less differentiated subsets (data not shown). We suggest that a loss of mitochondrial mass in the CD8^+^ EMRA subsets is a key mediator in generating an enhanced senescent state.

We then examined differences in mitochondrial respiration between the CD4^+^ and CD8^+^ EMRA subsets. Differences were found in both the baseline respiration and respiration following injection of oligomycin, FCCP and rotenone and antimycin A (Figure 2e). The CD4^+^ EMRA population retain their ability to respond to challenge akin to the other CD4^+^ memory subsets (Figure S3e), while we have shown this not to be the case for the CD8^+^ EMRA compartment (Henson et al., 2014). The oxygen consumption rate (OCR), together with the spare respiratory capacity, the potential amount of stored energy a cell has to respond to challenge were both upregulated in CD4^+^ EMRAs, further implying a difference in mitochondrial content. While the extracellular acidification rate (ECAR), a marker of lactic acid production and glycolysis, was only marginally increased compared to the CD8^+^ EMRA subset (Figure 2f), furthermore, the amount of ATP made by CD4^+^ EMRAs was also greater than that of the CD8^+^s (Figure 2g). These results suggest that the CD4^+^ EMRA subset has enhanced mitochondrial fitness that allows for a greater flexibility in the type of metabolism they can engage.

### 2.3 CD8^+^ EMRA T cells display impaired nutrient uptake

**Figure 3:**
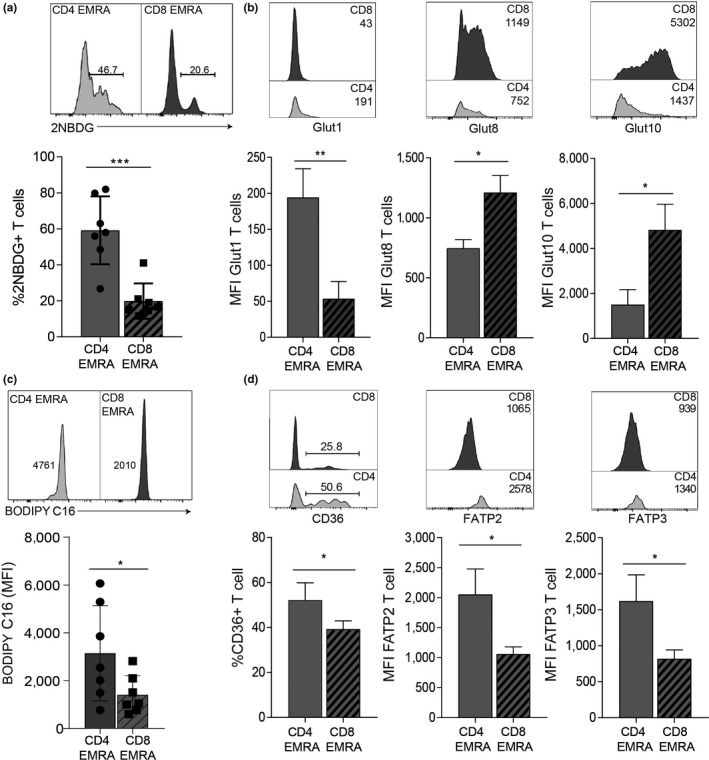
Impaired nutrient uptake by CD8^+^ EMRA T cells. (a) Glucose uptake was assessed using the fluorescent glucose analogue 2‐NBDG in CD4^+^ and CD8^+^ T CD45RA/CD27‐defined EMRA T cells from middle‐aged donors by flow cytometry following a 15‐min incubation. Data expressed as mean ± *SEM* of seven donors. (b) Examples and data showing expression of the glucose transporters glut1, glut8 and glut10 in senescent T cell subsets directly ex vivo from middle‐aged donors. Graphs show the mean ± *SEM* for four donors. (c) Lipid uptake was measured using fluorescently labelled palmitate, BODIPY C16 by flow cytometry following a 15‐min incubation in CD4^+^ and CD8^+^ EMRA T cells from middle‐aged donors. Data expressed as mean ± *SEM* of seven donors. (d) Examples and graphs showing the fatty acid translocase CD36 and FATP2 and −3 directly ex vivo from middle‐aged donors*.* Data expressed as mean ± *SEM* of six donors. *p*‐values were calculated using a *t* test. *p < .05, **p < .01, and ***p < .005

T cells utilize a variety of energy sources including glucose and lipids; however, their metabolic preferences are governed not only by their differentiation status but also by mitochondria fitness (Cui et al., 2012). Indeed, a lack of regulatory control over nutrient usage is a recurrent theme accompanying senescence and aging (Brewer, Gibbs, & Smith, 2016). We therefore sort to determine whether there were differences in glucose and fatty acid uptake in CD4^+^ and CD8^+^ EMRA T cell subsets. CD4^+^ EMRAs were found to take up more of the fluorescent glucose analogue 2‐NBDG from their extracellular environment than their CD8^+^ EMRA counterparts (Figure 3a). This was found to be independent of the age of the donor (Figure S4a). Indeed, CD4^+^ EMRAs showed higher expression of glut1, the major glucose transporter in T cells using an RNA‐labelled probe (Figure 3b). Analysis of microarray data revealed high expression of alternate glut family members (Callender et al., 2018). Interestingly, CD8^+^ EMRAs displayed a higher expression of the class III glucose transporters glut8 and glut10 (Figure 3b). Both these transporters are found intracellularly and are thought to transport glucose or galactose across intracellular organelle membranes (Mueckler & Thorens, 2013). The uptake of fluorescently labelled palmitate, BODIPY C16, a long‐chain fatty acid, was also quantified. Similar to our observations for glucose uptake, CD4^+^ EMRA T cells also utilize significantly more palmitate than their CD8^+^ counterparts (Figure 3c), again independent of the chronological age of the donor (Figure S4b). Furthermore, CD4^+^ EMRA T cells express higher levels of both the fatty acid translocase CD36 and the fatty acid transporters FATP2 and FATP3 (Figure 3d). Taken together, these results suggest that the increased mitochondrial fitness of CD4^+^ EMRA T cells enables these cells to better utilize glucose and lipids, which may limit the impact of senescence.

### 2.4 Impaired proliferation and migration of CD8^+^ EMRA T cells

**Figure 4:**
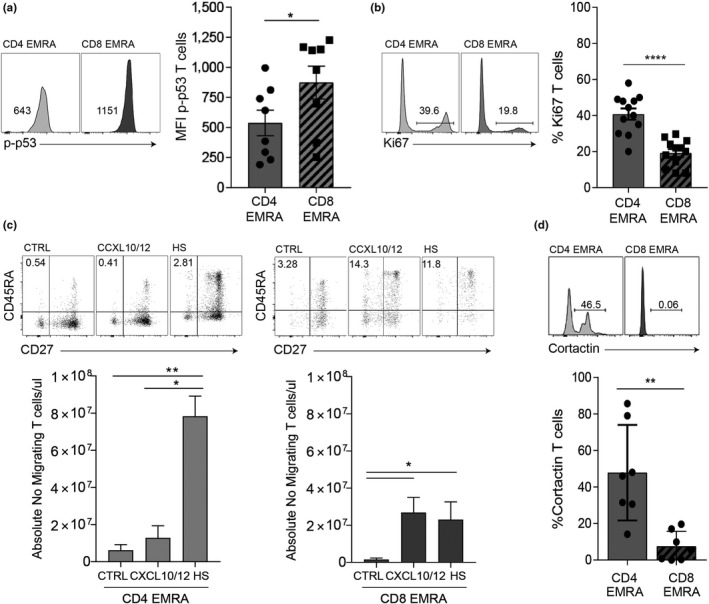
Impaired function observed in CD8^+^ EMRA T cells. (a) Example and graph showing the expression of p‐p53 in CD4^+^ and CD8^+^ CD45RA/CD27‐defined EMRA T cells directly ex vivo from middle‐aged donors. Graphs show the mean ± *SEM* for four donors. (b) Proliferation was defined in senescent T cell subsets using Ki67 directly ex vivo from middle‐aged donors*.* Data show the mean ± *SEM* for 12 donors. (c) The migration of CD4^+^ and CD8^+^ EMRA T cells from middle‐aged donors through HUVECs and their supporting transwell filters. HUVECs were stimulated with 20% decomplemented (heated at 56°C for 20 min) autologous donor sera for 24 hr. PBMCs were allowed to adhere and migrate for 4 hr towards either media, CXCL10/12 or autologous serum. The number of T cells was counted and expressed as a percentage of the total migrated CD4^+^ or CD8^+^ T cells. Data are expressed as the mean ± *SEM* of six donors. (d) Representative flow cytometry plots and cumulative graphs showing the percentage of cortactin‐positive senescent T cell subsets analysed directly ex vivo from middle‐aged donors. Data expressed as mean ± *SEM* of seven donors. *p*‐values were calculated using a *t* test. *p < .05, **p < .01, ***p < .005, and ****p < .001

The end result of the DNA damage response is the activation of p53. p53 regulates cell cycle arrest limiting cell growth and proliferation, as well as playing a crucial role in limiting cell motility, a critical process for optimal T cell function (Muller, Vousden, & Norman, 2011). In line with the theory that the acquisition of the CD4^+^ EMRA T cell subset occurs at a slower rate than their CD8^+^ counterpart, we find that the expression of p‐p53 is higher in the CD8^+^ EMRAs compared to the CD4^+^s (Figure 4a), although the expression of p‐p53 in the CD4^+^ EMRAs is the highest of all the CD4^+^ memory subsets (data not shown). Furthermore, the proliferative defect is more pronounced in the CD8^+^ EMRA subset, measured using ki67 (Figure 4b) and migration impaired (Figure 4c). Transwell chemotactic assays were used to assess migration; HUVECs were activated using 20% autologous donor sera, in order to create a more appropriate ex vivo environment, and were found to be no different to activation with IFNγ (Figure S4c). Migration was assessed in response to CXCL10 and CXCL12, chemokines promoting the migration of memory T cells or 20% autologous serum, and the data expressed as a percentage of the total CD4^+^ or CD8^+^ T cells found to have migrated. CD4^+^ EMRA T cells were less able to respond to CXCL10 and CXCL12 than autologous serum, presumably due to the loss of CXCR3 and CXCR4 from the CD4^+^ EMRA T cells (Brainard et al., 2007; Hess et al., 2004). CD8^+^ EMRAs on the other hand retain expression of CXCR3 and CXCR4 and migrate in response to both chemokines and autologous serum, all be it to a lesser extent than their CD4^+^ counterparts (Figure 4c). This was once again not dependent on the chronological age of the donors (Figure S4d).

The enhanced migratory capacity of the CD4^+^ EMRA subset was also evident in their enhanced cortactin expression (Figure 4d). Cortactin is known to mediate complex roles in cell migration and invasion (Kirkbride, Sung, Sinha, & Weaver, 2011), where it is involved in the formation of lamellipodia and invadopodia (Murphy & Courtneidge, 2011). Furthermore, the loss of p53 has been shown to promote invasion (Wang, Zhang, Kong, Zhang, & Zhu, 2013). Taken together with the findings that p53 inhibits mitochondrial biogenesis, CD4^+^ EMRAs potentially retain more functionality than their CD8^+^ counterpart, as they are better able to undergo the necessary metabolic reprogramming needed to generate effector functions owing to their higher mitochondrial mass.

### 2.5 Impairing mitochondrial function in CD4^+^ T cells accelerates senescence

**Figure 5:**
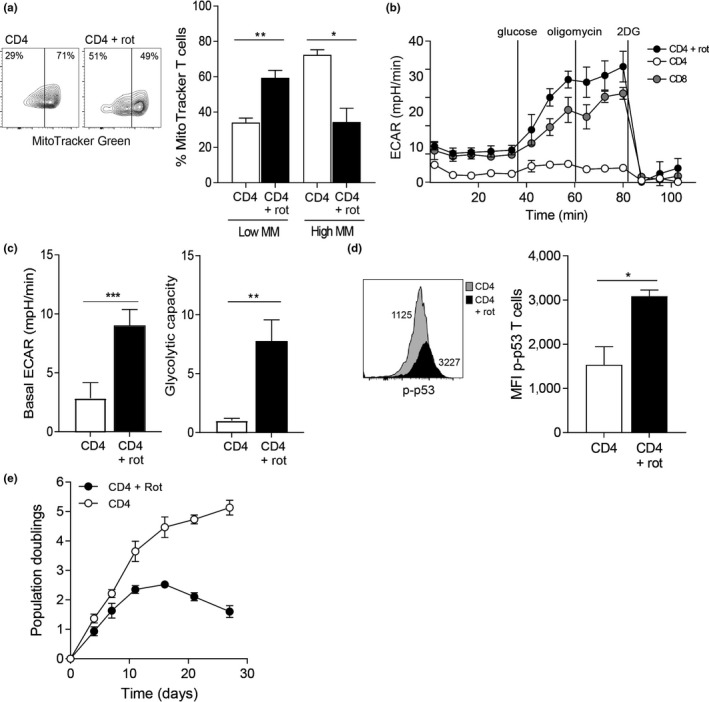
Impairing mitochondrial function in CD4^+^ T cells accelerates senescence. (a) Example and graph showing the mitochondrial mass of CD4^+^ T cells treated for 5 days with 10 nM rotenone or DMSO control. Graph shows the mean ± *SEM* for three middle‐aged donors. (b) Extracellular acidification rates (ECAR) of the rotenone‐ or DMSO‐treated CD4^+^ T cells or DMSO‐treated CD8^+^ T cell from middle‐aged donors were measured following a 15‐min stimulation with 0.5 µg/ml anti‐CD3 and 5 ng/ml IL‐2. The cells were then subjected to a glycolytic rate assay using the indicated substances. Data are representative of three independent experiments. (c) The basal ECAR and glycolytic capacity were measured following a 15‐min stimulation with 0.5 µg/ml anti‐CD3 and 5 ng/ml IL‐2. Graphs show the mean ± *SEM* for three donors. (d) Example and graph showing the expression of p‐p53 in CD4^+^ T cells from middle‐aged donors treated for 5 days with 10 nM rotenone or DMSO control. Graphs show the mean ± *SEM* for three donors. (E) Population doublings for CD4^+^ T cells treated 10 nM rotenone or DMSO control over 27 days. Graphs show the mean ± *SEM* for three donors

We then wanted to investigate whether impairing mitochondrial function in CD4^+^ T cells could induce a similar phenotype observed in the CD8^+^ compartment. We used rotenone, a complex I inhibitor, to damage mitochondria. After a 5‐day treatment with a low‐dose (10 nM) rotenone, we observed an increased amount of low mass mitochondria at the expense of the higher more functional fused mitochondria in CD4^+^ T cells (Figure 5a). The change in mitochondrial state leads to a switch in metabolism, with the rotenone‐treated CD4^+^ T cells showing a strong response to the addition of glucose akin to the CD8^+^ T cells (Figure 5b). The rotenone treatment increased the basal glycolysis levels as well as increasing the glycolytic capacity of CD4^+^ T cells (Figure 5c). There is growing evidence that p53 can also regulate mitochondrial function, maintaining mitochondrial respiration through the transactivation of SCO2 (synthesis of cytochrome c oxidase 2; Matoba et al., 2006). However, stress conditions led to the translocation of p53 from the nucleus to the mitochondria leading to mitochondria‐mediated apoptosis (Qi et al., 2011). We observe here that treatment of CD4^+^ T cells with rotenone leads to high levels of phospho‐p53 (Figure 5d) and a slowing in cell growth that eventually leads to a loss of CD4^+^ T cells (Figure 5e), therefore leading us to conclude that the higher mitochondrial mass observed in CD4^+^ T cells is protective against senescence by maintaining an oxidative state.

## 3 DISCUSSION

Immunosenescence is a hallmark feature of aging and is accompanied by a chronic low‐grade inflammatory state. Together, these features are important drivers in numerous age‐related pathologies (Baker et al., 2011). Senescent or EMRA T cells are a highly dynamic and heterogeneous subset of cells that accumulate with age (Callender et al., 2018) and are found in both the CD4^+^ and CD8^+^ T cell compartments. We and others have shown the CD8^+^ EMRA subset to accumulate more rapidly than their CD4^+^ counterparts with age (Akbar et al., 2016; Czesnikiewicz‐Guzik et al., 2008; Koch et al., 2008). Both subsets undergo the same phenotypic and functional changes, showing loss of co‐stimulatory molecules and the acquisition of NK cell markers, together with a reduction in proliferative capacity and changes in cytokine production. It has been suggested that the CD4^+^ EMRA subset is more resistant to the effects of age owing to better homeostatic control in this compartment compared to that of CD8^+^ T cells. However, we show here that it is enhanced mitochondrial dysfunction in the CD8^+^ EMRA subset that alters metabolic stability that governs the susceptibility of an immunosenescent state.

Mitochondrial dysfunction is a central event in many pathologies and contributes to age‐related processes. Mitochondria have been shown to participate in every aspect of aging, such as a decline in stem cell function, cellular senescence and the development of the low‐grade inflammatory state (Theurey & Pizzo, 2018). Alterations that occur to mitochondria with age are numerous, including reductions in mitochondrial mass (Corsetti et al., 2008), defects in mitochondrial biogenesis (Reznick et al., 2007) and impaired mitochondrial function in terms of ATP production and respiratory chain capacity (Preston et al., 2008). Indeed, we show here that the CD8^+^ EMRA T cell subset displayed a lower mitochondrial mass and impaired mitochondrial biogenesis, as well as having a hypopolarized mitochondrial phenotype compared to the CD4^+^ EMRA subset. When taken together, these data indicate that the CD8^+^ EMRA subsets have a greater degree of mitochondrial impairment than the CD4^+^ compartment. We do not wish to oversimplify the idea that the presence of highly active mitochondria increases senescence resistance, as studies have demonstrated that mild reduction of mitochondrial function can counterintuitively increase lifespan in lower organisms (Theurey & Pizzo, 2018). However, the overexpression of mitochondrial enzymes in yeast increases lifespan and caloric restriction (Kaplon et al., 2013), a well‐established process to increase lifespan, and mediates its effects through improved mitochondrial activity. Therefore, we believe that the enhanced mitochondrial function observed for the CD4^+^ EMRA T cell subset does confer a survival advantage at the cellular level.

Increases in ROS levels have been demonstrated to be critical for the induction and maintenance of cell senescence (Davalli, Mitic, Caporali, Lauriola, & D'Arca, 2016). However, ROS are also secondary messengers in cellular antioxidant pathways and rather than being thought of as deleterious by‐products and can be beneficial through the induction of an adaptive response that counteracts the rise in oxidative stress (mitohormesis; Ristow & Zarse, 2010). Indeed, low levels of ROS have been implicated in improved cellular fitness and lifespan extension in various animal models (Owusu‐Ansah, Song, & Perrimon, 2013). The mitochondrial membrane potential (ΔΨm) is the central bioenergetic parameter controlling the generation of ROS (Nicholls, 2004). We show here that the CD8^+^ EMRA subset displayed a hypopolarized phenotype whereas the CD4^+^ EMRAs were in a hyperpolarized state, with both subsets showed very little protein leak. As expected from cells that display a high ΔΨm, CD4^+^ EMRAs also produced more ROS than their CD8^+^ counterparts. However, the higher mitochondrial content observed in CD4^+^ EMRA T cells means these cells have more buffering capacity to quench the effects of the ROS. We postulate that the CD4^+^ EMRAs are better at controlling the damaging effect of ROS and are therefore better able to control the rate of senescence. Similar mechanisms have been reported in hepatocytes, whereby a reduction in mitochondrial ROS production, through a decrease in proton leak, and a higher ΔΨm were found to be beneficial (Divakaruni & Brand, 2011). However, it remains to be established what role uncoupling proteins (UCPs) play in mitigating ROS production by senescent T cells. Mouse models have shown a positive correlation between increased uncoupling and lifespan (Speakman et al., 2004). We can only infer a potential role for UCP1. UCP1 is activated by fatty acids, and we show here that the CD4^+^ EMRA population take up more fatty acids and retain more fatty acid transporters than the CD8^+^ EMRA subset.

Mitochondria also play a key role in cellular metabolism, and they house the electron transport chain and the TCA cycle, as well as playing crucial roles in the synthesis and breakdown of lipids (Pence & Yarbro, 2018). Metabolic regulation plays an important role during cellular senescence, with dysregulated metabolism now identified as a feature of many different cell types including T cells (Henson et al., 2014). There is increasing evidence that cell cultures become glycolytic as they age, depending on the senescence induction method used (James et al., 2015). Indeed, we have shown previously that CD8^+^ EMRA T cells as they differentiate lose their metabolic plasticity and become more glycolytic (Henson et al., 2014). However, we show here that CD4^+^ EMRAs retain their metabolic flexibility showing better glycolytic and oxidative capacity than their CD8^+^ counterparts. Furthermore, they also retain better glucose and lipid uptake together with increased expression of transporters. Interestingly, CD8^+^ EMRAs displayed increased expression of the glucose transporters glut8 and glut10. It is tempting to speculate in the light of a recent publication showing that glut6 functions as a glycolysis modulator in inflammatory macrophages without influencing glucose uptake (Maedera et al., 2019) and that glut8 and glut10 may act in a similar manner in senescent CD8^+^ T cells. Glut6, glut8 and glut10 are all members of the Glut III family of transporters, with glut6 and glut8 being very closely located on chromosome 9 and glut10 having a very high affinity for both 2‐deoxy‐D‐glucose and D‐galactose (Zhao & Keating, 2007); both slow the rate of glycolysis. However, further work needs to be carried out in order to establish whether glut8 and glut10 play a negative role in glucose uptake on T cells.

Metabolic reprogramming is necessary in order for T cells to fulfil their function. Upon activation, CD4^+^ and CD8^+^ T cells can differentiate into a diverse array of effector populations, with memory cells using fatty acid oxidation, while effectors are glycolytic (van der Windt Gerritje et al., 2012). CD4^+^ T cells can further differentiate into subsets with effector or suppressor roles. The in vitro generation of Th1, Th2 and Th17 CD4^+^ T cell subsets was all found to utilize glycolysis, whereas T regulatory cells favoured mitochondrial oxidative pathways (Michalek et al., 2011). However, both CD4^+^ and CD8^+^ EMRA T cells are effectors, predominantly polarized towards Th1 (Sakata‐Kaneko, Wakatsuki, Matsunaga, Usui, & Kita, 2000; Saurwein‐Teissl et al., 2002) and should have similar metabolisms. It has been demonstrated that metabolic reprogramming is fine‐tuned by co‐stimulatory signals, in particular CD28, the signals from which cause an increase in mitochondrial mass (Klein Geltink et al., 2017). It is therefore tempting to speculate that the mitochondrial differences observed between CD4^+^ and CD8^+^ EMRA lie in the fact that CD8^+^ EMRAs lose CD28 first and then CD27 while CD4^+^ EMRAs lose CD27 and then CD28 (Koch et al., 2008).

The changes that occur to nutrient usage during senescence impact T cell function, as is evident from the accelerated loss of function seen in CD8^+^ EMRA T cells. Senescent CD8^+^ T cells showed greater impairment in proliferative capacity than their CD4^+^ EMRA counterparts, in part, due to their reduced ability to take up and efficiently utilize metabolites. However, the phenotype seen in CD8^+^ EMRA T cells also mirrors that reported for haematopoietic cells undergoing ER stress: compromised mitochondrial function, lower ATP levels and reduced glut1 expression (Wang et al., 2011). Cell metabolism also exerts a strong influence on T cell migration (Buck et al., 2016). The improved mobilization of energy substrates by CD4^+^ EMRA T cells allows them to be better equipped to deal with the high‐energy demands of the migration process. Interestingly, the CD4^+^ EMRAs were unable to respond to CXCL10 and CXCL12, as the expression of CXCR3 and CXCR4 has both been shown to decrease with differentiation in the CD4^+^ subset but is retained by CD8^+^s (Brainard et al., 2007; Hess et al., 2004). However, chemokine receptor expression in CD4^+^ T subsets does not universally decline, as the CD4^+^ EMRA subset shows robust transmigration in response to autologous serum. The migratory advantage of CD4^+^ EMRA T cells may also be explained by their increased expression of cortactin, a core element of T cell locomotion involved in the formation of lamellipodia and invadopodia (Murphy & Courtneidge, 2011). Taken together, the greater loss in proliferative and migratory capacity of senescent CD8^+^ T suggests a potential greater impairment to CD8^+^ immunity. Indeed, failure to produce an antibody response following flu vaccination has been associated with an increase in senescent CD8^+^ T cells (Saurwein‐Teissl et al., 2002).

We postulate here that mitochondrial density influences the extent of T cell senescence in a p53‐dependent manner. Studies have shown that p53 can influence mitochondrial function, and under steady‐state conditions, p53 maintains mitochondrial respiration through the regulation of SCO2 (Matoba et al., 2006). SCO2 is critical for regulating the cytochrome c oxidase complex, the major site of oxygen utilization. However, under stress settings, p53 functions to control mitochondrial quality through the overexpression of SCO2, ROS generation and the removal of mitochondria (Qi et al., 2011) via mitophagy or a protease‐dependent degradation of damaged proteins (Dai et al., 2016). We show here that the relatively lower expression of p53 in CD4^+^ versus CD8^+^ EMRA T cells maintains an oxidative metabolism, and increasing mitochondrial stress in the CD4^+^ T cells leads to a more CD8^+^‐like glycolytic metabolism that is accompanied by increased apoptosis. Our work goes against findings in fibroblasts where the targeted depletion of mitochondria through the impairment of their biogenesis demonstrated that decreased numbers of mitochondria were able to prevent the senescence response (Correia‐Melo et al., 2016). It is possible that for T cells, therapies aimed at increasing mitochondrial mass would be beneficial in combating the detrimental effects of senescence during aging.

Collectively, our results suggest that mitochondrial mass controls the senescence phenotype in T cells. However, the mechanism remains elusive, and it is not via a DNA damage response that has been suggested by others (Correia‐Melo et al., 2016). Mitochondria are a central part in inducing and governing the rate of T cell senescence. Therefore, identifying T cell‐specific therapies aimed at increasing mitochondrial mass would be beneficial in combating the detrimental effects of senescence during aging.

## 4 EXPERIMENTAL PROCEDURES

### 4.1 Blood sample collection, isolation and cell culture

Heparinized peripheral blood samples were taken from healthy volunteers, with average age of 41 years ± 5. Healthy volunteers were taken as individuals who had not had an infection or immunization within the last month, no known immunodeficiency or history of chemotherapy or radiotherapy, and were not receiving systemic steroids within the last month or any other immunosuppressive medications within the last 6 months. PBMCs were isolated using Ficoll‐Hypaque (Amersham Biosciences). All samples were obtained in accordance with the ethical committee of Royal Free and University College Medical School and the North East—York Research Ethics Committee 16/NE/0073. Human umbilical vein endothelial cells (HUVECs) were cultured according to the supplier's instructions (PromoCell).

### 4.2 Flow cytometric analysis and cell sorting

Flow cytometric analysis was performed using the following antibodies: CD4 PECF594 (RM4‐5) from BD Biosciences and CD8 PerCP (SK1), CD45RA BV605 (HI100), CD27 BV421 (O323), CD28 BV785 (CD28.2), CCR7 PECy7 (G043H7) and CD36 APCCy7 (5‐271) from BioLegend. FATP2 (Abcam) and FATP3 (Atlas antibodies) were measured in conjunction with goat anti‐rabbit AF488 (Abcam). Cortactin expression was assessed using rabbit anti‐human cortactin antibody (PA5‐27134; Life Technologies) stained in conjugation with goat anti‐rabbit Cy3 (Life Technologies). PGC1α (3G6) and p‐p53 (16G8) both from Cell Signaling, and Ki67 (B56; BD Bioscience) were assessed by intracellular staining using solution AB (Thermo Fisher) and goat anti‐rabbit AF488 (Abcam). All samples were run using an LSR II (BD Biosciences) and analysed using FlowJo software (Treestar).

Magnetic beads were used to isolation of CD8^+^ and CD4^+^ T cells (Miltenyi Biotec) according to the manufacturer's instructions. The purity of T cell subsets was assessed by flow cytometry.

### 4.3 PrimeFlow RNA assay

PrimeFlow RNA Assay technology was used to determine SLC2A gene expression according to the manufacturer's instructions (Thermo Fisher). PBMCs were incubated with the following gene‐specific probes: SLC2A10 (AF647—Type 1 probe set), SLC2A8 (AF488—Type 4 probe set) and SLC2A1 (AF750—Type 6 probe set). Samples were analysed immediately as described above.

### 4.4 Proliferation assays

CD45RA/CD27‐sorted CD4^+^ and CD8^+^ T cells were stimulated with 0.5 µg/ml plate coated with anti‐CD3 (OKT3) and 5 ng/ml IL‐2 for 3 days, and proliferation was assessed by staining for the cell cycle‐related nuclear antigen Ki67 as described above.

### 4.5 Transmission electron microscopy studies

CD27/CD45RA‐defined CD4^+^ and CD8^+^ EMRA T cell subsets were isolated and fixed in 2% paraformaldehyde and 1.5% glutaraldehyde in 0.1 m phosphate buffer pH 7.3. They were then osmicated in 1% OsO4 in 0.1 M phosphate buffer, dehydrated in a graded ethanol–water series, cleared in propylene oxide and infiltrated with Araldite resin. Ultrathin sections were cut using a diamond knife, collected on 300 mesh grids and stained with uranyl acetate and lead citrate. The cells were viewed in a JEOL 1010 transmission electron microscope (Jeol) and imaged using a Gatan Orius CCD camera (Gatan). Mitochondrial volume density (percentage of T cell volume occupied by mitochondria) was determined from EM images using a point‐counting method using ImageJ.

### 4.6 Mitochondrial measurements

Mitochondrial mass was assessed by incubating labelled PBMCs with 100 nM of MitoTracker Green FM (Thermo Fisher) for 30 min at 37°C, 5% CO_2_. Mitochondrial membrane potential was investigated using TMRE (Thermo Fisher), 1 µM TMRE was incubated with labelled PBMCs for 30 min at 37°C, 5% CO_2_. Mitochondrial ROS was measured using MitoSOX (Thermo Fisher), 2 µM MitoSOX was incubated with labelled PBMCs for 20 min at 37°C, 5% CO_2_. Unfixed samples were immediately collected on a LSR II (BD Bioscience).

### 4.7 ATP determination

Intracellular ATP levels were measured ex vivo on sorted CD4^+^ and CD8^+^ T cell subsets via a bioluminescence assay according to the manufacturer's instructions (Thermo Fisher).

### 4.8 Metabolic assays

Oxygen consumption rates and ECAR were measured in CD45RA/CD27‐sorted CD4^+^ and CD8^+^ T cell subsets following 15‐min stimulation with 1 µg/ml anti‐CD3 and 5 ng/ml IL‐2. The assay was performed in RPMI without phenol red and carbonate buffer (Sigma) containing 25 mM glucose, 2 nM L‐glutamine and 1 mM pyruvate. The metabolic stress test was performed using 1 µM oligomycin, 1.5 µM fluorocarbonyl cyanide phenylhydrazone (FCCP), 100 nM rotenone and 1 µM antimycin A (Sigma) with the XF‐96 Extracellular Flux Analyzer (Agilent). Glycolysis was examined via ECAR in assay buffer excluding glucose, following injection with 10 mM glucose, 1 µM oligomycin and 100 mM 2‐deoxy‐D‐glucose (2‐DG; Sigma).

### 4.9 Glucose and lipid uptake assays

To assess glucose and lipid uptake in T cell subsets, PBMCs were incubated with anti‐CD3 (1 µg/ml) for 30 min at 37°C. Cells were subsequently incubated with 1 nM Bodipy FL C_16_ or 100 µM 2‐NBDG, both from Thermo Fisher in PBS and incubated for 15 min in media containing no glucose or serum. Samples were then analysed by flow cytometry.

### 4.10 Transwell migration assay

HUVECs monolayers were grown to confluence on transwell membranes (Corning) in the presence of 20% autologous donor sera or 10 ng/ml IFNγ (R&D Systems). PBMCs from healthy donors were placed in M199 medium (Sigma) in the top well and the chemoattractant in the bottom well, which was either 20% autologous donor sera or 1 g/ml CXCL10 and CXCL12 (R&D Systems). Cell migration was assessed after incubation at 37°C for 4 hr; each condition was set up in duplicate transwells. Migrated T cells were then collected from the top and bottom wells, respectively, stained with phenotypic markers and quantified for a fixed period of time (3 min) by flow cytometer. Counting beads (BD Bioscience) were also run to enumerate the total number of cell to have transmigrated.

### 4.11 Rotenone cultures

Purified CD4^+^ T cells were incubated for 5 days with 10 nM rotenone (Sigma) or DMSO control after which time the cells were used for metabolic assessment: mitochondrial mass, p53 levels and extracellular flux analysis as describe above. For long‐term rotenone cultures, 10 nM rotenone was plated together with 0.2 × 10^6^ CD4^+^ T cells and 0.5 µg/ml anti‐CD3 and 5 ng/ml IL‐2. Cells were counted every 4/5 days, and population doubling was calculated using the following equation: PD = log10(Nf/Ni)/log(2), Nf = number of cells harvested, Ni = initial cell number seeded.

### 4.12 Statistical analysis

GraphPad Prism was used to perform statistical analysis. Statistical significance was evaluated using the paired Student *t* test. Differences were considered significant when *p* was < .05.

## CONFLICT OF INTEREST

The authors have no conflicting financial interests.

## AUTHOR CONTRIBUTIONS

SMH wrote the paper, designed and performed the experiments, and analysed the data. LAC, ECC and EAB performed experiments. ES and ANA designed experiments as well as reviewing the manuscript.

## Data Availability

The data that support the findings of this study are available from the corresponding author upon reasonable request.
