# Supplementary figures and images for: Mitochondrial mass governs the extent of human T cell senescence
Source: Aging Cell. 2019 Dec 2;19(2):e13067. doi: 10.1111/acel.13067 (PMC6996952; doi:10.1111/acel.13067)

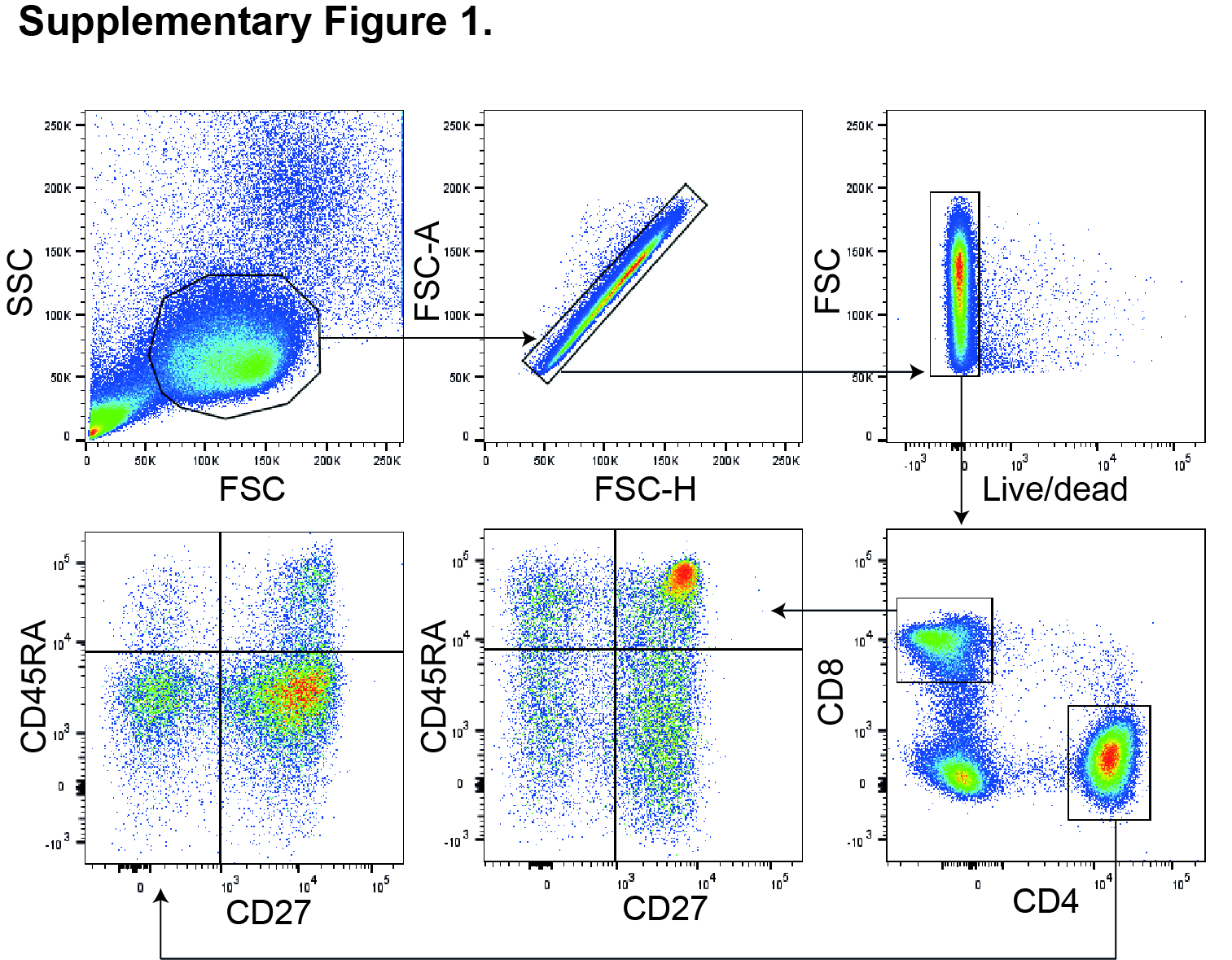

Supplement: Supplementary file 1 [file ACEL-19-e13067-s001.jpg]

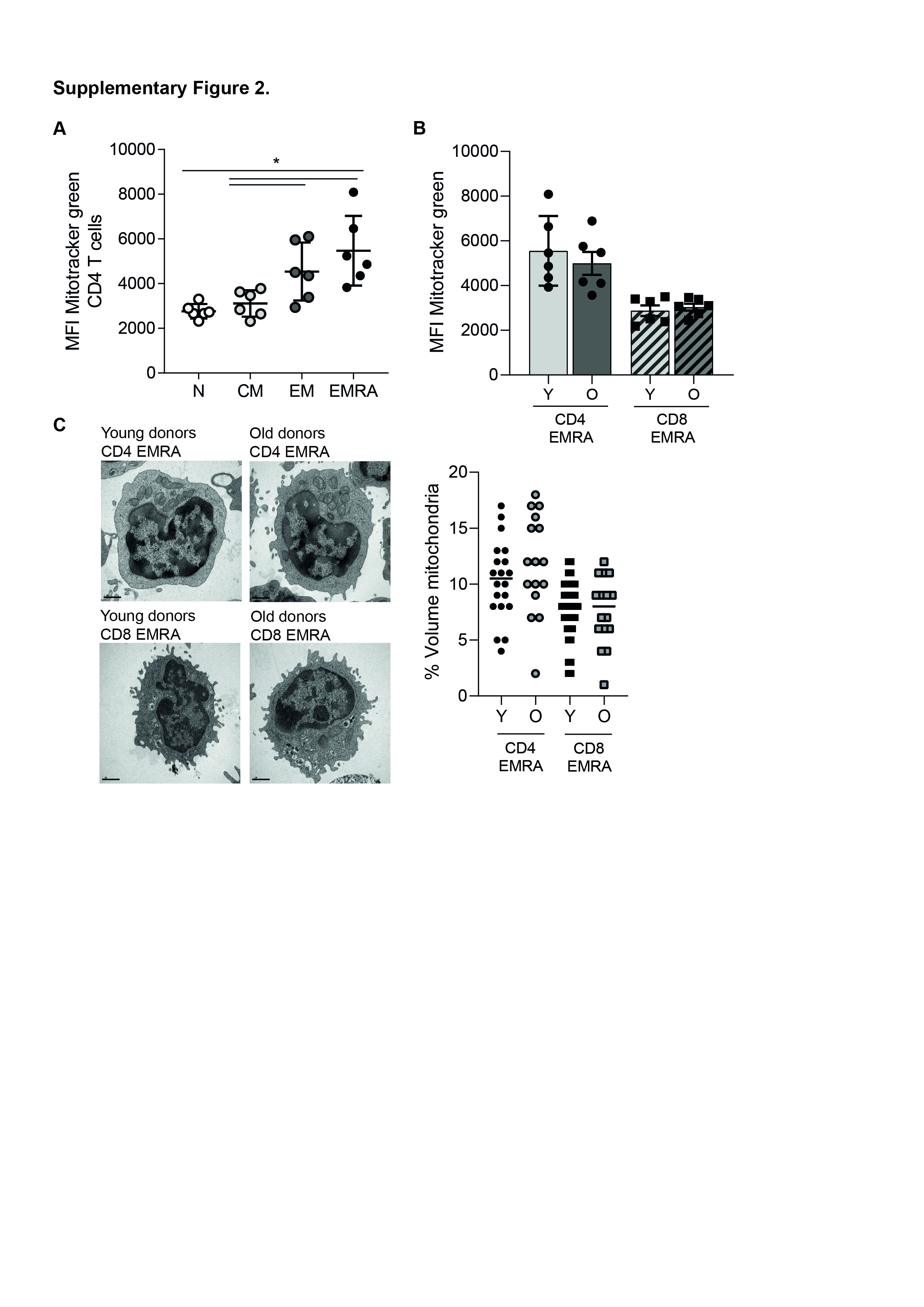

Supplement: Supplementary file 2 [file ACEL-19-e13067-s002.jpg]

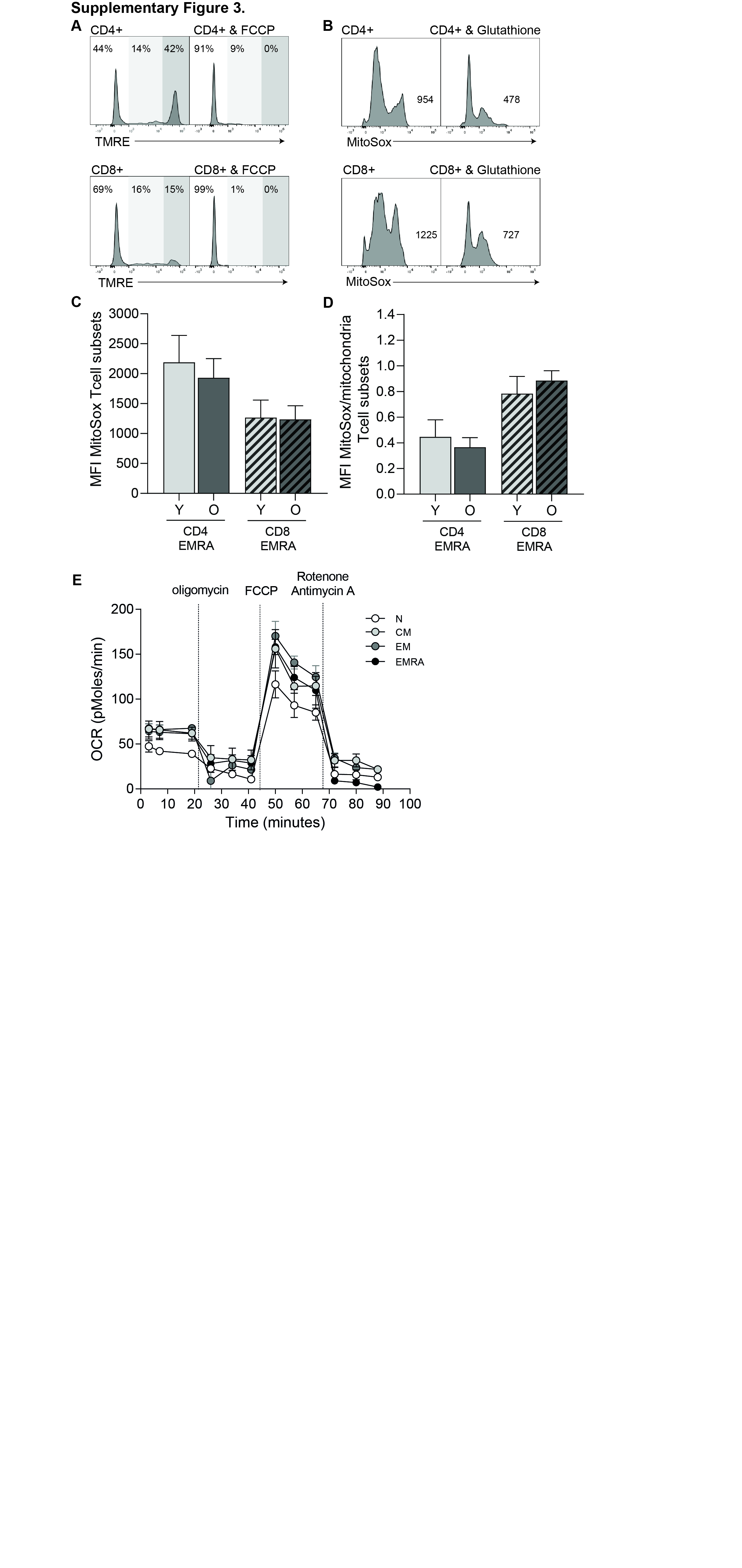

Supplement: Supplementary file 3 [file ACEL-19-e13067-s003.jpg]

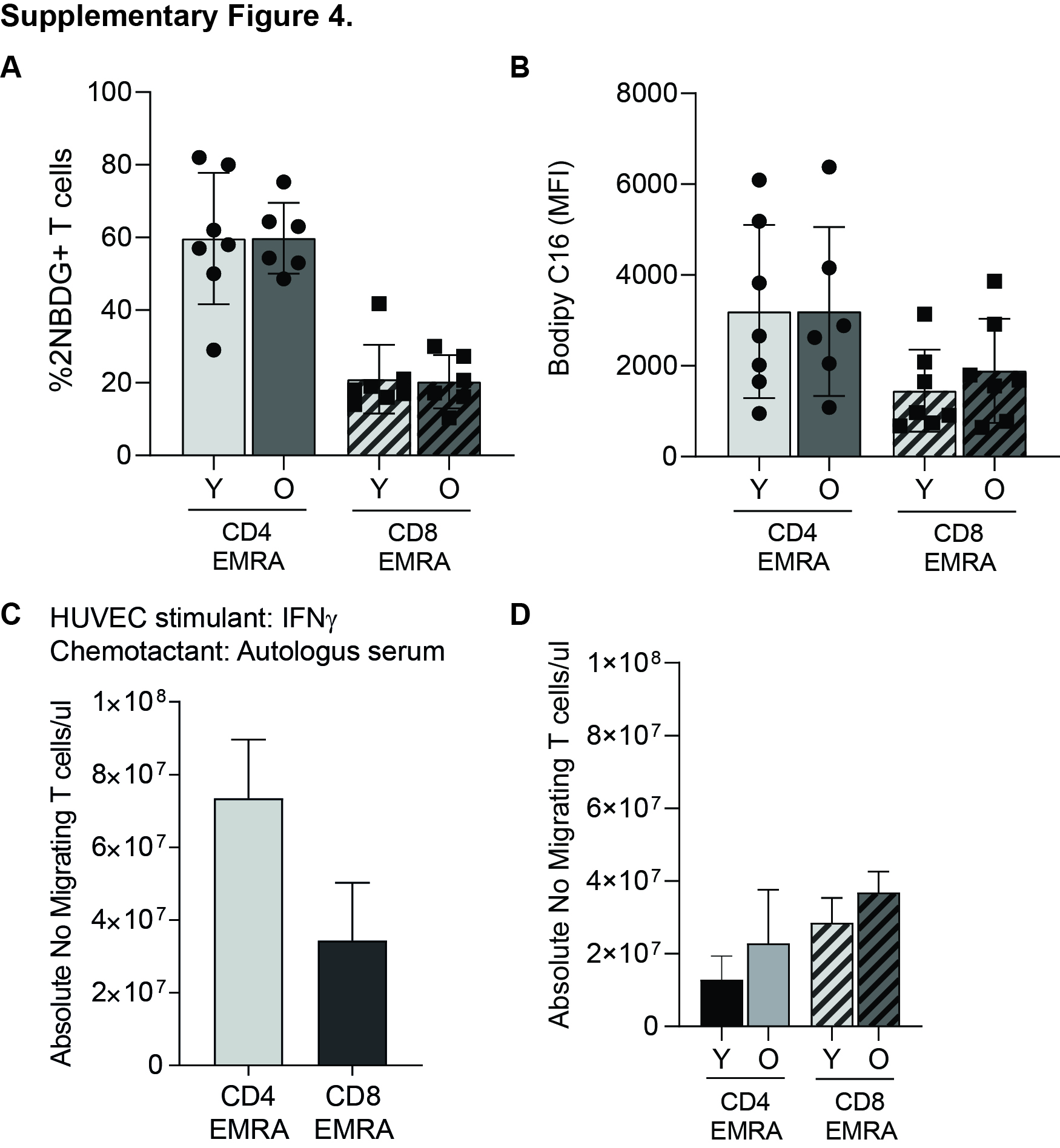

Supplement: Supplementary file 4 [file ACEL-19-e13067-s004.jpg]
