# Supplementary material for: Mitochondrial mass governs the extent of human T cell senescence
Source: Aging Cell. 2019 Dec 2;19(2):e13067. doi: 10.1111/acel.13067 (PMC6996952; doi:10.1111/acel.13067)
Supplement: Supplementary file 5 [file ACEL-19-e13067-s005.docx]

**Supplementary figure legends**

**Supplementary Figure 1. Experimental gating strategy**

All experiments were first gated on the lymphocyte population, followed by live cells then the CD4^+^ or CD8^+^ population before the CD45RA/CD27 profile was obtained.

**Supplementary Figure 2. Comparison of mitochondrial mass in EMRA T cells isolated from young and old donors.**

A) Mitotracker green staining in CD4^+^ CD27/CD45RA defined T cells from young middle aged donors analysed directly *ex vivo*. Data expressed as mean ± SEM of 6 donors. B) Comparison of mitotracker green staining in CD4^+^ and CD8^+^ EMRA T cells isolated from young (average age 41 years ± 5) and old individuals (average age 68 years ± 2) analysed directly *ex vivo*. Data expressed as mean ± SEM of 6 donors. (C) Comparison of electron microscope images of CD4^+^ and CD8^+^ EMRA T cells isolated from young and old individuals imaged directly *ex vivo.* Graph shows the percentage by cell volume of mitochondria in senescent T cell subsets.

**Supplementary Figure 3. Comparison of mitochondrial function in EMRA T cells isolated from young and old donors.**

A) TMRE staining of CD4^+^ or CD8^+^ T cells with 20µM FCCP included as a negative control. B) MitoSox staining of CD4^+^ or CD8^+^ T cells with 10µM glutathione as a negative control. C) Comparison of MitoSox staining in CD4^+^ and CD8^+^ EMRA T cells isolated from young (average age 41 years ± 5) and old individuals (average age 68 years ± 2) analysed directly *ex vivo*. Data expressed as mean ± SEM of 6 donors. D) Mitochondrial ROS production expressed as a ratio of mitochondrial mass in young and old donors. Calculated from data shown in supplementary fig 1B and supplementary fig 2B. E) Oxygen consumption rates (OCR) of CD27/CD45RA defined CD4^+^ T cells were measured following a 15 minute stimulated with 0.5 µg/ml anti-CD3 and 5 ng/ml IL-2, the cells were then subjected to a metabolic stress test using the indicated mitochondrial inhibitors. Data are representative of 4 independent experiments using cells isolated from middle aged donors.

**Supplementary Figure 4. Comparison of nutrient uptake and function in EMRA T cells isolated from young and old donors.**

A) Comparison of glucose uptake assessed by 2-NBDG uptake in CD4^+^ and CD8^+^ EMRA T cells isolated from young (average age 41 years ± 5) and old individuals (average age 68 years ± 2). Data expressed as mean ± SEM of 7 young donors and 6 old donors. B) Comparison of lipid uptake measured using fluorescently labelled palmitate, BODIPY C16 in CD4^+^ and CD8^+^ EMRA T cells isolated from young (average age 41 years ± 5) and old individuals (average age 68 years ± 2). Data expressed as mean ± SEM of 7 young donors and 6 old donors. C) The migration of CD4^+^ and CD8^+^ EMRA T cells was also tested through HUVECs that had been stimulated with 10 ng/ml IFNγ rather than autologous donor sera. PBMCs were allowed to adhere and migrate for 4h towards autologous serum. The number of T cells were counted and expressed as a percentage of the total CD4^+^ or CD8^+^ T cell subset added. Data are expressed as the mean ± SEM of 4 donors. D) Comparison of the migratory capacity of CD4^+^ and CD8^+^ EMRA T cells isolated from young (average age 41 years ± 5) and old individuals (average age 68 years ± 2) through HUVECs in response to CXCL10/12. The number of T cells were counted and expressed as a percentage of the total CD4^+^ or CD8^+^ T cell subset added. Data are expressed as the mean ± SEM of 6 donors.
